# Pregnancy outcomes in women with a systemic right ventricle and transposition of the great arteries results from the ESC-EORP Registry of Pregnancy and Cardiac disease (ROPAC)

**DOI:** 10.1136/heartjnl-2020-318685

**Published:** 2021-04-28

**Authors:** Oktay Tutarel, Lucia Baris, Werner Budts, Mohamad Gamal Abd-El Aziz, Csilla Liptai, David Majdalany, Silvana Jovanova, Alexandra Frogoudaki, Heidi M Connolly, Mark R Johnson, Aldo P Maggioni, Roger Hall, Jolien W Roos-Hesselink, the ROPAC Investigators Group, Christopher Peter Gale

**Affiliations:** 1 Department of Congenital Heart Disease and Paediatric Cardiology, German Heart Centre Munich, TUM School of Medicine, Technical University of Munich; DZHK (German Centre for Cardiovascular Research), partner site Munich Heart Alliance, Munich, Germany; 2 Department of Cardiology, Erasmus Medical Center Rotterdam, Rotterdam, The Netherlands; 3 Department of Cardiology, University Hospital Leuven, Leuven, Belgium; 4 Cardiology, CCU, Assiut General Hospital, Assiut, Egypt; 5 Department of Cardiology, Semmelweis University Medical Center, Budapest, Hungary; 6 Department of Cardiology, Cleveland Clinic, Cleveland, Ohio, USA; 7 Department of Cardiology, University Clinic of Cardiology, University t Cyril and Methodius, Skopje, North Macedonia; 8 Department of Cardiology, Adult Congenital Heart Clinic, Attikon University Hospital, Athens, Greece; 9 Department of Cardiology, University of Rochester Medical Center, New York, New York, USA; 10 Department of Obstetric Medicine and Gynaecology, Imperial College London, Chelsea and Westminster Hospital, London, UK; 11 EURObservational Research Programme, European Society of Cardiology, France and Maria Cecilia Hospital, GVM Care & Research, Cotignola, Italy; 12 Department of Cardiology, University of East Anglia Norwich Medical School, Norwich, UK

**Keywords:** transposition of great vessels, pregnancy

## Abstract

**Objective:**

Cardiac disease is a major cause of maternal mortality. Data regarding pregnancy outcomes in women with a systemic right ventricle (sRV) are scarce. We studied pregnancy outcomes in women with an sRV after the atrial switch procedure for transposition of the great arteries (TGA) or congenitally corrected TGA (CCTGA).

**Methods:**

The ESC EORP Registry of Pregnancy and Cardiac Disease is an international prospective registry of pregnant women with cardiac disease. Pregnancy outcomes (maternal/fetal) in all women with an sRV are described. The primary end point was a major adverse cardiac event (MACE) defined as maternal death, supraventricular or ventricular arrhythmias requiring treatment, heart failure, aortic dissection, endocarditis, ischaemic coronary event and other thromboembolic events.

**Results:**

Altogether, 162 women with an sRV (TGA n=121, CCTGA n=41, mean age 28.8±4.6 years) were included. No maternal mortality occurred. In 26 women, at least one MACE occurred, heart failure in 16 (9.8%), arrhythmias (atrial 5, ventricular 6) in 11 (6.7%) and others in 4 (2.5%). Prepregnancy signs of heart failure as well as an sRV ejection fraction <40% were predictors of MACE. One woman experienced fetal loss, while no neonatal mortality was observed. No significant differences were found between women with CCTGA and TGA. In the subset of women who had an echocardiogram before and after pregnancy, no clear deterioration in sRV was observed.

**Conclusion:**

The majority of women with an sRV tolerated pregnancy well with a favourable maternal and fetal outcome. Heart failure and arrhythmias were the most common MACE.

## Introduction

In patients with transposition of the great arteries after the atrial switch procedure (TGA), the morphological right ventricle (RV) acts as the systemic ventricle. The same holds true for patients with congenitally corrected transposition of the great arteries (CCTGA). Ongoing concerns have been expressed about the long-term ability of the systemic RV (sRV) to support systemic pressure and its capacity to handle the volume load of pregnancy.^1 2^ However, there is a paucity of information about pregnancy outcomes in women with an sRV.^1 3^ Retrospective studies, in the majority from single centres, provided conflicting information.^4 5^ Large prospective studies are lacking. Yet, these studies are needed to provide evidence for guidelines on the management of pregnancy in women with an sRV and to counsel women who are contemplating pregnancy. The aim of this study is to assess in a prospective worldwide study, maternal and fetal outcomes of pregnancy in women with an sRV.

## Methods

### Study design

The European Society of Cardiology (ESC) EURObservational Research Programme (EORP) Registry on Pregnancy and Cardiac disease (ROPAC) is an international, prospective, observational registry of pregnant patients with structural or ischaemic heart disease, aortic pathology and pulmonary arterial hypertension. Study design and methods have been described in detail previously.^6^

ROPAC was initiated by the ESC working groups on congenital heart disease and valvular heart disease in 2007 and embedded in the EORP of the ESC. Pregnant patients were included prospectively from 2007 and for this analysis we included all pregnancies in patients with an sRV enrolled between January 2007 and January 2018. Women with a univentricular circulation were excluded.

### Data

The ROPAC study protocol and the first results of this registry were published in 2013.^6^ Patients with a diagnosis of TGA or CCTGA were identified from the registry. Baseline characteristics collected before pregnancy included age, New York Heart Association (NYHA) functional classification, ECG rhythm, diagnosis, risk factors (smoking habits, hypertension, diabetes), medication, previous interventions, parity and obstetric history and echocardiographic measurements. Information regarding the ejection fraction (> or <40%) of the sRV—which was assessed by echocardiography—was mandatory, while additional information, for example, tricuspid regurgitation, was collected, but was not mandatory. Countries were divided into high-income or emerging countries according to the International Monetary Fund Classification.^7^

### Definitions and end points

The primary combined end point was the occurrence of a major adverse cardiac event (MACE), defined as combined end point of maternal death, supraventricular or ventricular arrhythmias requiring treatment, heart failure, aortic dissection, endocarditis, ischaemic coronary event and other thromboembolic events. The secondary end points were adverse obstetric outcomes and adverse fetal/neonatal outcomes. Heart failure was defined according to the American College of Cardiology/American Heart Association guidelines,^8^ and heart failure episodes were only included when they required hospital admission, new treatment or change in the existing treatment regimen. Impaired systemic ventricular function was defined as an sRV ejection fraction <40%. Postpartum haemorrhage was defined as increased blood loss during delivery up to 24 hours post partum requiring specific interventions. Haemolysis, elevated liver enzymes, low platelets (HELLP) syndrome, pre-eclampsia and eclampsia and pregnancy-induced hypertension were defined according to the International Society for the Study of Hypertension in Pregnancy 2012 statement.^9^ Fetal mortality was defined as the death of a fetus after 20 weeks of gestation until birth. Neonatal mortality was defined as the death of a live-born baby in the first 6 months of life. Premature birth was defined as birth before 37 weeks of gestation. Low birth weight was defined as a birth weight of <2500 g. Low Apgar score was defined as an Apgar score at 5 min of <7. All outcomes were examined for the duration of the pregnancy and up to 6 months post partum.

### Patient and public involvement

This research was done without patient involvement.

### Statistical analysis

Data are presented as mean values and SD if normally distributed and median with IQR if skewed. Categorical data are presented as frequencies and percentages. Baseline characteristics and outcomes were compared between women with TGA and CCTGA with χ^2^ tests, Fisher’s exact tests, Student’s t-tests and Mann-Whitney U tests where appropriate. Comparisons between prepregnancy and postpartum echocardiograms were performed with the McNemar’s test. Univariable analyses to identify baseline characteristics associated with outcomes were performed. Predictors used were the prepregnancy variables: age, parity, diagnosis, CCTGA, country, signs of heart failure, NYHA class >1, sRV end-diastolic diameter >42 mm, sRV ejection fraction <40% and pulmonary hypertension. Missing values were handled with multiple imputation. A p value of <0.05 (two-sided test) was considered significant. All statistical tests and analyses were performed with SPSS V.21.0 (SPSS, Chicago, Illinois, USA).

## Results

Of the 5739 patients included in the ROPAC registry from January 2007 to January 2018,^10^ 162 women had an sRV (TGA after atrial switch n=121 and CCTGA n=41). None of the CCTGA patients had a history of tricuspid valve surgery. Mean age was 28.8±4.6 years, and 86 women (52.8 %) were primigravida. Baseline characteristics are presented in table 1 for the whole cohort and for TGA and CCTGA patients, respectively. Most of the women were asymptomatic or had only mild symptoms (NYHA class I/II) before pregnancy. Only one woman in the TGA group was in NYHA class III. Women with TGA received cardiac medication before pregnancy in 30.6% and those with CCTGA in 39%. Outcomes of pregnancy are presented in tables 2 and 3. Maternal mortality did not occur during pregnancy or up until 6 months postdelivery. Of all patients with an sRV, hospital admission for a cardiac reason was required in 9.8% during pregnancy. This occurred more often in women with CCTGA than TGA (19.5% vs 6.6%, p=0.03). The main reason for hospital admissions was heart failure for both groups. The majority of heart failure episodes occurred in the second and third trimester (9/16 events), while one took place in the first trimester. Post partum, six women had heart failure episodes (three in the first week post partum, two within 1 month and one within 6 months). Out of these, three already had heart failure episodes during pregnancy. At baseline, sinus rhythm was present in 123 patients (76%), while a pacemaker rhythm was reported in 14 (8.6%). Out of these 14, 7 had a CCTGA. Of the 14 women with pacemaker rhythm, 3 hospital admissions for cardiac reasons occurred (1 for heart failure during pregnancy and 2 for arrhythmias). There was no fetal death in this subgroup, however, six preterm deliveries occurred, of which two had a low Apgar score. Supraventricular tachycardia occurred in five (3.1%) patients, and ventricular tachycardia in six (3.7%) patients.

**Table 1:** Baseline characteristics

	Systemic RV All (n=162)	CCTGA (n=41)	TGA after atrial switch (n=121)	P value
Age in years (mean, SD)	28.8 (4.6)	28.0 (5.9)	29.1 (4.0)	0.22
Living in an emerging country	15 (9.2)	8 (19.5)	7 (5.8)	0.02
Primigravida	86 (52.8)	22 (53.7)	64 (52.9)	1.00
Current smoking	7 (5.0)	2 (5.4)	5 (5.0)	1.00
Prior diabetes mellitus	2 (1.2)	0 (0)	2 (1.7)	1.00
Prior hypertension	2 (1.2)	1 (2.4)	1 (0.8)	0.43
Signs of heart failure before pregnancy	8 (4.9)	3 (7.3)	5 (4.1)	0.42
Cardiac medication before pregnancy	53 (32.5)	16 (39.0)	37 (30.6)	0.34
Systemic RV dilatation	41 (25.3)	13 (31.7)	28 (23.1)	0.30
Systemic RV ejection fraction <40%	44 (27.0)	9 (22.0)	35 (28.9)	0.42
Pulmonary hypertension	4 (2.5)	1 (2.4)	3 (2.5)	0.99

Values are n (%) if not otherwise stated. P value for comparison between CCTGA and TGA.

CCTGA, congenitally corrected transposition of the great arteries; RV, right ventricle.

**Table 2:** Maternal outcomes of pregnancy

	Systemic RV All (n=162)	CCTGA (n=41)	TGA after atrial switch (n=121)	P value
Maternal mortality ≤6 months post partum	0 (0)	0 (0)	0 (0)	n.a.
Hospital admission for a cardiac reason	16 (9.8)	8 (19.5)	8 (6.6)	0.03
Heart failure	16 (9.8)	5 (12.2)	11 (9.1)	0.56
Supraventricular tachycardia	5 (3.1)	0 (0)	5 (4.1)	0.33
Ventricular tachycardia	6 (3.7)	2 (4.9)	4 (3.3)	0.64
Thromboembolic events	3 (1.8)	2 (4.9)	1 (0.8)	0.16
Endocarditis	1 (0.6)	1 (2.4)	0 (0)	0.25
Pregnancy-induced hypertension	7 (4.3)	2 (4.9)	5 (4.1)	1.00
(Pre-)eclampsia or HELLP	3 (1.8)	0 (0)	3 (2.5)	0.57
Postpartum haemorrhage	11 (6.7)	3 (7.3)	8 (6.6)	1.00

Values are n (%).

CCTGA, congenitally corrected transposition of the great arteries; HELLP, haemolysis elevated liver enzymes low platelet count; n.a., not available; RV, right ventricle.

**Table 3:** Obstetric and fetal outcomes of pregnancy

	Systemic RV All (n=162)	CCTGA (n=41)	TGA after atrial switch (n=121)	P value
Caesarean section	79 (48.5)	25 (61.0)	54 (44.6)	0.07
Of which for cardiac reasons	26 (32.9)	14 (56.0)	12 (22.2)	0.01
Emergency caesarean section for cardiac reasons	6 (3.7)	3 (7.3)	3 (2.5)	0.17
Fetal death	1 (0.6)	0 (0)	1 (0.8)	1.00
Neonatal death	0 (0)	0 (0)	0 (0)	n.a.
Premature birth	34 (21.0)	8 (20.0)	26 (21.5)	1.00
IUGR	7 (4.3)	1 (2.4)	6 (5.0)	0.68
Low Apgar scores	12 (7.4)	3 (7.3)	9 (7.4)	1.00
Small for gestational age	29 (17.8)	7 (17.1)	22 (18.2)	1.00

Values are n (%).

CCTGA, congenitally corrected transposition of the great arteries; IUGR, intrauterine growth retardation; n.a., not available; RV, right ventricle.

Both, a prepregnancy and postpartum echocardiogram with information of RV dimensions was present in 40 women. At prepregnancy, 27 out of 40 women had a dilated RV (end-diastolic diameter >40 mm). At the postpartum echocardiogram, 31 women had a dilated RV (p=0.34). Out of the group with a dilated RV, three improved, while seven with a normal diameter prepregnancy deteriorated to the dilated group. Information regarding tricuspid regurgitation (TR) at prepregnancy was available in 64 women (CCTGA n=17, TGA 47). Out of the CCTGA group, 3 women (17.6%) had no or mild TR, while 14 (82.4%) had moderate-to-severe TR. These numbers were 33 (70.2%) and 14 (29.8%) for the TGA group, respectively (p=0.01 for CCTGA vs TGA). Prepregnancy and postpartum information regarding TR were available in 46 women. Out of these at prepregnancy no or mild TR was present in 28, and moderate-to-severe TR in 18. Post partum these numbers were 29 and 17, respectively. Two women out of the no or mild TR group deteriorated to moderate TR, while in three women with moderate TR at prepregnancy no or mild TR was present post partum.

A caesarean section was performed in 46.7% of the whole ROPAC cohort compared with 48.5% of women with an sRV.^10^ It was more common with 61% in women with CCTGA compared with 44.6% of women with TGA, although not statistically significant (p=0.19). Of the 79 women who underwent caesarean section, 26 did so because of cardiac reasons. Most common reasons were heart failure (n=6, 23%), severity of cardiac disease (n=9, 34%), dysrhythmias (n=4, 15%) and anticoagulation use (n=2, 8%). Fetal death was not reported in women with CCTGA, and occurred in one woman with TGA, while it occurred in 1.3% of all women included in ROPAC.^10^ There were 34 premature births, out of these 15 were induced labours and 12 spontaneous (7 unknown). Preterm birth was not significantly associated with maternal heart failure (p=0.15) or higher NYHA class (p=0.16), but was significantly associated with maternal (cardiac) medication use (p=0.01). Of the 34 women who delivered preterm, 10 were using cardiac medication during pregnancy compared with 10 of the 116 who did not deliver preterm (online supplemental table 1). In 12 women data on prematurity was missing. Women with an sRV gave birth to babies with low birth weight in 17.8% vs 11.7% for the whole ROPAC cohort.^10^ Low birthweight infants were born to women with CCTGA in 17.1% vs 18.2% in women with TGA (p=0.87).

10.1136/heartjnl-2020-318685.supp1 Supplementary data

### Predictors of adverse outcomes

**Figure 1:**
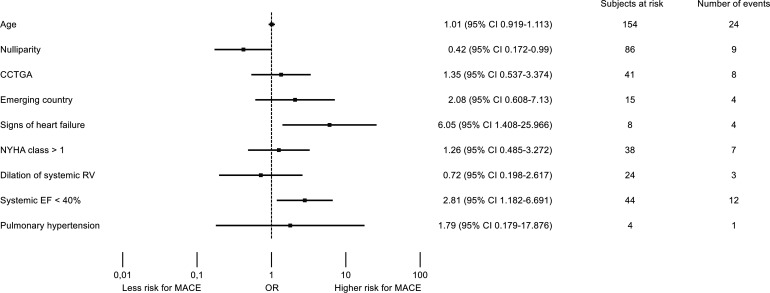
Predictors of adverse maternal outcome. Forest plot illustrating the results of the univariate logistic regression analysis for adverse maternal outcome, defined as MACE. CCTGA, congenitally corrected transposition of the great arteries; EF, ejection fraction; MACE, major adverse cardiac events; NYHA, New York Heart Association; RV, right ventricle.

Results of univariable logistic regression analyses are presented in figure 1. Prepregnancy signs of heart failure as well as a RV ejection fraction <40% were predictors of MACE, while being primigravida reduced the risk.

## Discussion

**Figure 2:**
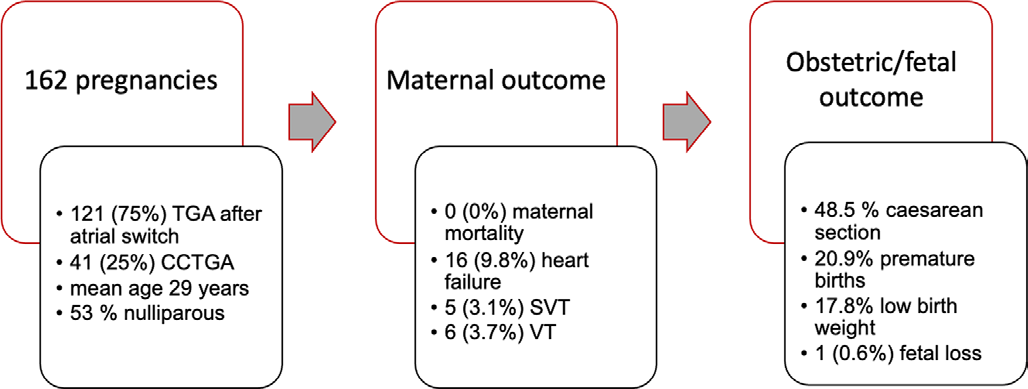
Maternal and fetal outcome. CCTGA, congenitally corrected transposition of the great arteries; SVT, supraventricular tachycardia; VT, ventricular tachycardia.

This international prospective registry studies the outcomes of pregnancy in women with an sRV. Our contemporary data show that a majority of women with an sRV tolerate pregnancy well with relatively low rates of MACE and without maternal or neonatal mortality (figure 2). Fetal mortality occurred in only one (0.6%) patient. Still, heart failure occurred in approximately 10% of women during pregnancy, while arrhythmias were observed in 6.7%. With the exception of hospital admissions for cardiac reasons, which were more common in women with CCTGA, there was no significant difference regarding maternal or fetal outcome between women with CCTGA and TGA.

### Maternal outcome

The results of this first large prospective study support the data from the few retrospective studies that reported no maternal mortality related to pregnancy in women with an sRV.^4 5 11^ Only one study of 70 pregnancies in 40 women reported severe sRV failure leading to cardiac transplantation after delivery in one woman, and another case in which the woman developed heart failure and then died suddenly 1 month after delivery.^12^ In this later study, there was a higher number of patients with a decreased RV function prior to pregnancy compared with our cohort.^12^ A number of complications such as arrhythmias, heart failure and symptomatic baffle obstructions have been described in women with an sRV during pregnancy.^5 13^ Most frequently, arrhythmias and heart failure are encountered with a rate between 7%–22% and 7%–21%, respectively, depending on the study design and patient population.^5 12–14^ In our contemporary registry, these numbers were lower with arrhythmias occurring in around 7% of women and heart failure in 10%. When comparing the cohorts from previously published reports with our cohort, striking differences are not present. On univariate analysis, predictors of MACE were signs of heart failure before pregnancy as well as an sRV ejection fraction <40%. In most of the previous studies due to the small number of women included or the retrospective design, predictors of adverse maternal outcome were not reported. However, the numbers in our study are too small to perform multivariate analysis.

### Obstetric and fetal outcome

In our contemporary cohort, both obstetric and fetal complications were less frequent than reported by previous studies.^5 12^

The most frequent observed fetal morbidity was premature birth (20.9%), followed by low birth weight (17.8%). While one fetal loss was observed, there was no neonatal mortality. These numbers compare favourably with pregnancy outcomes of other cardiac disease like hypertrophic cardiomyopathy or aortic stenosis.^15 16^ Likewise, they are lower than those reported by a study from the Dutch ZAHARA registry, where premature birth was observed in 31.4%, and being small for gestational age in 21.6%.^5^ Likewise, a multicentre, retrospective study from the USA reported rates of 31% for low birth weight, and 39% for premature birth.^12^ Fetal and neonatal mortality was also higher in the Dutch registry (combined 11.8%).^5^

Pregnancy-induced hypertensive disorder occurred in seven women (4.3%), while HELLP or (pre-)eclampsia was observed in three (1.8%). In the study by Drenthen *et al*,^5^ hypertensive disorders were more common (18.4%), including cases of pre-eclampsia and HELLP (10.2%). Similarly, Canobbio *et al* observed hypertensive disorders in 17% of women.^12^

A caesarean section was performed in 48.5% of women with an sRV in our study. Whether the (high) rates in our study are attributable to women having an sRV is not certain, but it is probable that women with heart disease in general are handled with more care and apprehension by treating physicians and are thus given a caesarean section (as many physicians prefer this apparently more controlled environment). Furthermore, the use of caesarean section as primary mode of delivery is very country and centre dependent.

### CCTGA versus TGA after atrial switch

No significant differences in pregnancy outcome were found between women with CCTGA and TGA in the current study. Only hospital admission for a cardiac reason occurred more often in women with CCTGA than TGA (19.5% vs 6.6%, p=0.03). Published data comparing pregnancy outcomes between women with CCTGA and TGA are scarce. A retrospective study from London/UK reported the results of 14 women with an sRV, of which 11 had TGA and 3 had CCTGA. Cardiac complications occurred only in women with TGA.^14^ Connolly *et al* reported the Mayo Clinic experience with pregnancy among women with CCTGA.^3^ Additionally, a more contemporary cohort from Poland was studied by Kowalik *et al*.^17^ Both reported a similar or more favourable outcome for women with CCTGA during pregnancy compared with reports of women with TGA.

Serial echocardiographic data were only available in a limited number of women. Therefore, analysis regarding the course and outcome of sRV ejection fraction was not possible. However, in 40 women with prepregnancy and postpartum sRV dimensions, 54% of those with a normal measurement at prepregnancy, dilated post partum. In a study by Cataldo *et al*, worsening of ventricular function was encountered in 29% of pregnant women with an sRV, of which 50% recovered during follow-up.^18^ An earlier study reported the echocardiographic data on the sRV dimensions in 18 pregnancies,^2^ 5 women (31%) experienced worsening of their RV dimensions during 7 pregnancies. While this progression in RV size was noted during or after a first pregnancy in four cases, it occurred after a second pregnancy in one, and a third pregnancy in two.^2^ Our results indicate that being primigravida reduced the risk of MACE. Maybe the sRV is able to tolerate the haemodynamic strain of one pregnancy but is less capable to tolerate the repeated strains posed by a second or third pregnancy. In addition, the sRV is known to deteriorate with age, and some of the observed deterioration with repeated pregnancy may be related simply to the passage of time.^19^ In one smaller study, the rate of deterioration was similar between women who became pregnant and those who did not after 8 years of follow-up.^18^ A larger comparative study of matched cohorts of women who have and have not undergone pregnancy over the same period would clarify whether pregnancy accelerates the rate of deterioration or not. Such a study would be particularly interesting with a long-term follow-up. An additional explanation besides the challenges of an sRV could be that peripartum cardiomyopathy also may play a role in some women.

The majority of the women with an sRV in our study had TGA. This is likely to change in the future, because the introduction of the arterial switch operation was a game changer for this population, which leads to a circulation, where the left ventricle is supporting the systemic circulation. Therefore, in the future most women with an sRV and a biventricular circulation will have CCTGA.

A limitation of our study is that serial echocardiographic data were only available in a limited number of women, and even in those was not complete. Therefore, analysis regarding the course and outcome of sRV ejection fraction as well as systemic atrioventricular valve regurgitation was not possible. But information regarding sRV dimensions was available. Additionally, all echocardiography parameters were obtained and assessed by the investigators from the including centre, inducing a possible interobserver variability for which we could not correct. Despite these limitations, this prospective registry included the largest number of women with sRV reported so far, providing important information related to the maternal and fetal outcome in women with an sRV.

## Conclusions

In conclusion, women with an sRV tolerate pregnancy surprisingly well with a favourable maternal and fetal outcome. Risk factors were prepregnancy signs of heart failure as well as an sRV ejection fraction <40%. Dedicated studies focusing on RV function and tricuspid valve regurgitation are warranted.

**Key messages**

**What is already known on this subject?**

- In patients with transposition of the great arteries after the atrial switch procedure as well as patients with congenitally corrected transposition of the great arteries, the morphological right ventricle acts as the systemic ventricle.
- Ongoing concerns have been expressed about the long-term ability of the systemic right ventricle to handle the volume load of pregnancy.
- There is a paucity of information about pregnancy outcomes in these women.

**What might this study add?**

- In this prospective, multicentre study on pregnancy outcomes in women with a systemic right ventricle, it was found that pregnancy is surprisingly well tolerated but that a systemic ejection fraction <40% and clinical signs of heart failure prior to pregnancy were risk factors of maternal complications during pregnancy.

**How might this impact on clinical practice?**

- Our results can reassure providers and patients with a systemic right ventricle that pregnancy is well tolerated if reduced ventricular function and clinical signs of heart failure are absent.

## Data Availability

All data relevant to the study are included in the article or uploaded as supplementary information. Data are owned by the ESC EURObservational Programme.
