# Supplementary material for: Pregnancy outcomes in women with a systemic right ventricle and transposition of the great arteries results from the ESC-EORP Registry of Pregnancy and Cardiac disease (ROPAC)
Source: Heart. 2021 Apr 28;108(2):117–23. doi: 10.1136/heartjnl-2020-318685 (PMC8717477; doi:10.1136/heartjnl-2020-318685)
Supplement: Supplementary data [file heartjnl-2020-318685supp001.pdf]

Supplemental Table 1. Cardiac medication use and preterm delivery

	**Preterm delivery**	**No preterm delivery**
All	34 (100%)	116 (100%)
Cardiac medication during pregnancy	10 (29%)	10 (9%)
Type of medication		
Statins	0 (0%)	0 (0%)
Diuretics	2 (20%)	0 (0%)
ACE-inhibitors	0 (0%)	0 (0%)
Beta blockers	7 (70%)	8 (80%)
Vitamin K antagonists	0 (0%)	0 (0%)
Anti-platelet therapy	2 (20%)	2 (20%)
